# Extraction Techniques and Analytical Methods for Characterization of Active Compounds in *Origanum* Species

**DOI:** 10.3390/molecules25204735

**Published:** 2020-10-15

**Authors:** Maša Knez Hrnčič, Darija Cör, Jana Simonovska, Željko Knez, Zoran Kavrakovski, Vesna Rafajlovska

**Affiliations:** 1 Laboratory for Separation Processes and Product Design, Faculty of Chemistry and Chemical Engineering, University of Maribor, Smetanova ulica 17, SI-2000 Maribor, Slovenia; darija.cor@um.si (D.C.); zeljko.knez@um.si (Ž.K.); 2 Department of Food and Biotechnology, Faculty of Technology and Metallurgy, Ss. Cyril and Methodius University in Skopje, Rudjer Boskovic 16, 1000 Skopje, Macedonia; jana@ukim.edu.mk (J.S.); vesna@ukim.edu.mk (V.R.); 3 Institute of Applied Chemistry and Pharmaceutical Analysis, Faculty of Pharmacy, Ss. Cyril and Methodius University in Skopje, Mother Theresa 47, 1000 Skopje, Macedonia; zoka@ukim.edu.mk

**Keywords:** oregano (*Origanum vulgare*), extraction, antioxidant activity, antimicrobial activity, phenolic compounds

## Abstract

Phytochemical research based on ethnopharmacology is gaining interest in industries such as functional food, nutraceuticals, cosmetics and pharmaceutical industries. Plants and plant extracts are a rich source of bioactive secondary metabolites. These compounds are often involved in plant protection against biotic or abiotic stresses. The exploitation of available technologies should be oriented and intensified to extend and enhance the continued usefulness of the plants as renewable sources of chemicals, especially medicinal compounds. This current contribution is focused on extraction and analytical techniques for their isolation from the oregano species, their characterization and their potential antioxidative, as well as their antimicrobial, antifungal and anticarcinogenic properties. The work is structured rendering to the different steps involved in the research; starting with extraction and sample preparation, followed by discussing the analytical techniques employed for the isolation and identification of compound/s responsible for the biological activity and methods and techniques for biological activity assessment.

## 1. Introduction

The Mediterranean Basin is an area representing only 1.6% of the Earth’s surface but is one of the world’s foremost centers for plant variety. Approximately 10% of the world’s higher plants can be found in this area [1]. It is home to a tremendous diversity of Mediterranean plants which are a rich source of phytochemicals and bioactive molecules important to human health. Aromatic plants play an important role in the discovery and development of new drugs, cosmetics and natural food supplements [2]. 

The genus *Origanum* (fam. *Lamiaceae*) is an aromatic perennial herb native to Western and Southwestern Eurasia and the Mediterranean region [3]. According to the classification of Ietswaart in 1980, it consists of 10 sections with 38 species, 6 subspecies and 17 hybrids. Since his publication to the present day, five new species and one hybrid have been identified [2]. The commercial name “oregano” is used for species that are rich in phenolic monoterpenes, carvacrol and thymol, while the name “marjoram” belongs to the species rich in bicyclic monoterpenoids cis- and trans-sabinene hydrate and cis-sabinene hydrate acetate [4]. 

The commercial oregano, (*Origanum vulgare*), also called *origanum* or wild marjoram is an aromatic perennial herb of the mint family (*Lamiaceae*) native to Western and Southwestern Eurasia and the Mediterranean region. The interest in its natural phytochemicals in relation to therapeutic and beneficial health properties has been increasing in recent years. For centuries, it has been used as a medicinal plant due to the several healthy properties, such as its powerful antioxidant, antibacterial and antifungal properties [5]. These valuable biocharacteristics of the plant’s extracts and essential oils, as well as its excellent adaptation to extreme drought and harsh environmental conditions, contributed to its attractiveness and implementation in agriculture ecosystems. The high value of *Origanum vulgare* is attributed to various extracts prepared from different parts of the plant, which chemically differ depending on extract preparation, origin of plant material (leaves, stems and flowers), developmental stage of the plant and plant taxonomy. Carvacrol and thymol are primary components of oregano essential oil, which are responsible for the antioxidant, antibacterial and antifungal actions [6]. 

Traditional extraction technologies have been well established but involve the application of considerable amounts of conventional solvents, such as alcohols, acetone, diethyl ether and ethyl acetate, while also using methods that are energy intensive, time consuming and influence the total process cost [7]. In applying the principles of green chemistry, water and carbon dioxide clearly represent safer solvents. 

The use of various gases in the liquid or supercritical state as extracting solvents has been under research for nearly half of a century. A large number of natural products have been extracted with carbon dioxide, and commercial plants in the food industry have already been well established. Supercritical fluid extraction (SFE) of oregano leaves is suggested to concentrate and isolate antioxidant extracts to be used as functional food ingredients. The extraction process has been evaluated in terms of both the quality and the activity of the antioxidant products obtained. LC–MS with electrospray has been used, along with a diode array detector, to characterize the extracts in terms of chemical composition; antioxidant activities have been measured using two different in vitro assays; 1,1-diphenyl-2-picrylhydrazyl (DPPH) antiradical test and bleaching β-carotene method.

The isolation and fractionation of phenolic compounds has been traditionally performed by well-established methods involving long processing times, high temperatures, large amounts of organic solvents and additional purification procedures for sample preparation, separation, detection and identification. However, these methods have been replaced lately by the advanced green techniques; amongst them SCE is the most frequently used. 

The decision on the extraction method employed is influenced by the chemical nature of the substance, sample particle size and by the presence of interfering substances. Extraction time, temperature, solvent-to-feed ratio, the number of repeated extractions of the sample and the choice of extraction solvents are the crucial parameters affecting the solubility, and consequently the yield, of the compound of interest. Phenolic compounds can be extracted from fresh, frozen or dried plant samples.

Before extraction, the material is pretreated by milling, grinding, drying and homogenization. The selection of the drying procedure impacts the total phenolic content. Freeze-drying retains higher levels of phenolic content in plant samples than air-drying [8].

**Figure 1:**
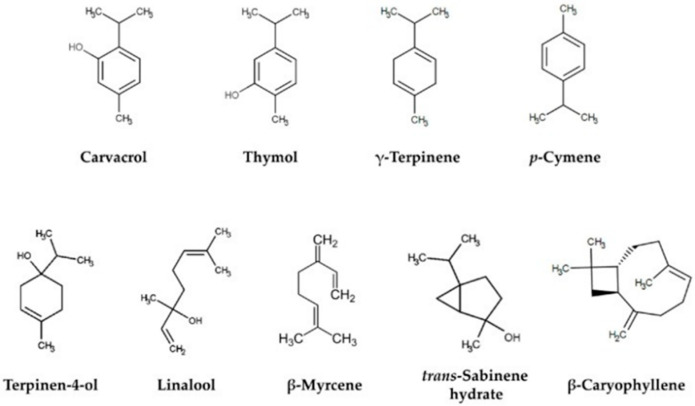
Some of the major constituents of the essential oils of oregano [9].

The solvent of choice must be featured with a low viscosity in order to accelerate mass transfer. At an elevated temperature, the viscosity of the solvent is reduced and the surface tension of the solvent is decreased, which results in a higher extraction rate. Extraction conditions are adjusted in order to obtain the highest yields of volatile substances, and the influence of the extraction conditions and the precision of the method are examined. Carvacrol, terpinen-4-ol, linalool, sabinene, α-terpinene and γ-terpinene (Figure 1) are the major components that contribute to antimicrobial and antioxidant activity [9].

## 2. Methods

### 2.1. Conventional Extraction Methods

The traditional methods of essential oil distillation are steam and hydrodistillation. Solid–liquid extraction, using solvents, such as methanol, ethanol and acetone is utilized for the extraction of antioxidants from plant matters. The extraction method and the extraction conditions can affect the chemical composition of the essential oils [10]. These effects need to be considered according to the application of essential oils [11]. Hydrodistillation is among the oldest extraction techniques for the isolation of essential oils. Long extraction times, which could lead to the hydrolysis of some heat-sensitive components, and complicated control of process parameters, which may consequence in inadequate extract composition, are the main disadvantages of the method. Therefore, researchers are chasing alternatives to this extraction principle [12]. The application of supercritical fluids as an extraction media could offer a solution to this issue. Indeed, according to the results of the evaluation and comparison of the chemical profile of essential oils of marjoram (*Origanum majorana*) and oregano (*Origanum vulgare*) obtained by hydrodistillation and supercritical CO_2_, both essential oils presented similar chemical profiles. Cis-sabinene hydrate was determined as the major compound in the essential oil obtained by supercritical extraction with CO_2_, whilst terpinen-4-ol was present as the main compound in the essential oil obtained by hydrodistillation. Supercritical CO_2_ extraction gave a higher yield of essential oils compared to hydrodistillation and the concentrations of oxygenated monoterpenes were higher without significant differences between fractions.

Therefore, selecting the appropriate method and the optimization of the extraction technique is among the important considerations in producing an extract with desirable characteristics. Advanced techniques have recently been proposed for the extraction of essential oils [13,14]. 

Supercritical fluid extraction is an efficient way to extract valuable constituents, suitable to replace the conventional methods [15,16] due to the absence of organic solvents and elevated temperatures that may lead to undesired reactions of active compounds such as hydrolyses, isomerization and oxidation, as well as inversely affect their antioxidant and antimicrobial characteristics.

When extracting active compounds from the original plant material, solvent extraction is frequently used. Cervato and co-workers reported a high proportion of antioxidant species in aqueous and methanolic extracts of oregano (*Origanum vulgare*) [17]. Different solvents and their mixtures have been applied for extraction of flavonoids and phenolic compounds from various oregano species.

Ethanol extraction of oregano leaves, yielding four compounds with strong antioxidant activity, has been reported by Chuang. Rosmarinic acid, quercetin, apigenin and carvacrol were identified by high-performance liquid chromatography [18]. Methanol has been the most widely applied solvent for the isolation of flavonoids and phenolic acids [19,20].

Methanol/water mixtures have been reported as effective extraction media for the isolation of caffeic acid glucoside, epigallocatechin, arbutin, luteolin rutinoside, luteolin glucuronide, rosmarinic acid, dihydroquercetin, dihydroluteolin, apigenin and quercetin [21,22,23,24].

Water has been proved as a proficient extraction solvent for the isolation of phenolic acids, including chlorogenic, vanillic, gallic, cinnamic, coumaric, ferulic and caffeic acid. In addition, chrysin, epicatechin, naringenin, catechin, kaempferol, quercetin, rutin, luteolin-7-*O*-glucoside, apigenin-7-*O*-glucoisde, rosmarinic acid and luteolin were also identified in the water extracts of various oregano species [25]. Chloroform [26], phosphate buffer [27] and acetone in mixtures with water and organic solvents have also been applied [28]. Reports have shown that flavones are among the most abundant subgroup of flavonoids in the extracts of oregano species followed by flavonols, flavanones and flavanol. The principal components of flavonoids and phenolic compounds may vary considerably in different oregano species. Additionally, geographical and environmental factors have been demonstrated as the key parameters determining the flavonoid and phenolic acids profile of chemotypes within the same species. Verbascoside has been identified in the water extract of the *Lippia citriodora* species [29], whilst caffeic acid, lithospermic acid and rosmarinic acid were present in the water extract of *Majorana hortensis* attained under the same process conditions [30]. Gallic acid, caffeic acid, 4-hydroxybenzaldehyde, *p*-coumaric acid and rosmarinic acid were present in the water extract of *Origanum acutidens* [31].

Thermal treatment causes the degradation of polyphenols or undesirable oxidation. Since the phenolic yield in the extracts is significantly decreased by the long-term thermal treatment, high processing temperatures should therefore be avoided. Temperatures exceeding 70 °C are not desired and lead to a rapid anthocyanin degradation. A long extraction time is yet another problem facing the conventional extraction procedure. In addition, maceration and Soxhlet extraction are the typical and most widely used conventional extraction methods and are known for their low efficiency and potential environmental hazard due to their high demand for organic solvents. Indeed, among innovative process technologies, supercritical CO_2_ extraction and fractionation is widely studied. Different authors compared supercritical extracts with those obtained by using liquid solvents (ethanol and hexane) or hydrodistillation, and described the superior functional activity of the supercritical extracts [32,33,34].

### 2.2. Extraction Methods Involving Sub- and Supercritical Fluids

Supercritical fluid extraction using carbon dioxide or subcritical water as a solvent meet the safety and environmental requirements and are therefore considered as clean and benign processes. Over steam distillation or conventional organic solvent extraction, extraction involving sub- and supercritical fluids, features several advantages. This includes the elimination of the organic solvents and the residue in the products and the capability to use lower temperatures, leading to less deterioration of the thermally-labile components in the extract. Supercritical fluid extraction of oregano leaves is suggested to concentrate and isolate antioxidant extracts to be used as functional food ingredients. Namely, the technique is suitable to retain the organoleptic characteristics of the starting spice materials. Compressed carbon dioxide yields a high-quality extract at a competitive cost. The pretreatment of the herbaceous matrices, the extraction pressure and temperature and the solvent flow rate [35] are among the main process parameters that affect the extraction kinetics and extract composition. In that sense, the extraction process has been evaluated in terms of both the quality and the activity of the antioxidant products obtained. Supercritical fluid extraction is also frequently more selective than extraction with commonly used solvents which extract unwanted components (e.g., tannins, chlorophyll and minerals). Some authors have reported on the supercritical extraction of oregano leaves to obtain oregano essential oil and oleoresin [36].

Subcritical water extraction has been utilized for the extraction of medically and commercially important phenolic compounds from food and food byproducts. Subcritical water extraction uses water at elevated pressures and temperatures to extract compounds of different polarities. Adjusting the water polarity is an additional advantage of this technique, which is achieved by changing the system temperature. The tuneability of operating conditions and consequent high selectivity of subcritical water extraction enables the handling of the composition of the extracts by varying the operating conditions and has therefore been used for essential oil isolation as well as for antioxidant extraction [37]. As reported by Rodríguez-Meizoso et al. [37], subcritical water extraction has been utilized for the extraction of nutraceuticals with antioxidant activity from oregano. The selectivity of the process has been investigated based on applying different operating conditions for the extraction of antioxidants from oregano leaves. Subcritical water extraction has been performed at different temperatures (25, 50, 100, 150 and 200 °C). The highest extraction temperature, 200 °C, gave the extracts of the highest antioxidant activity (EC50 equal to 10 μg/mL) and also the highest extraction yield (54% dry weight). Their contribution moreover strongly supports the theory that different temperatures involve extraction of different type of compounds.

### 2.3. Sub- and Supercritical Fluid Extraction by Adding Organic Modifiers

The drawback of the application of supercritical carbon dioxide as an extraction media is its low polarity. CO_2_ has a relatively low potency to extract polar compounds and is ought to be fortified by the addition of small amounts of organic modifiers. Leeke and co-workers have applied compressed carbon dioxide and CO_2_ plus water as an entrainer [38]. 

### 2.4. Ultra-Sound Assisted Extraction

Microwave-assisted extraction is simply a combination of microwave heating and a conventional extraction method such as solvent extraction and hydrodistillation. Power ultrasound induces phenomena in the medium, such as acoustic streaming and cavitation, which leads to intense agitation and mass transfer enhancement. Ultrasound-assisted extraction techniques therefore accelerate the release of the essential oils from aromatic plants as they facilitate the penetration of the solvents in the plant material [39]. Cavitation involves the formation, expansion and growth of small liquid-free zones or bubbles. These collapse sharply by producing mechanical forces, as well as local high temperatures and pressure at ambient conditions. Such behavior allows the release and dissolution of intracellular materials like essential oils. One of the recent applications of ultrasound is as part of the intensification of the supercritical fluid extraction of high added value compounds, such as antioxidants and essential oils. Ultrasound-assisted supercritical fluid extraction to obtain extracts rich in phenolic compounds with antioxidant capacity from dry oregano has been reported [40]. Whilst an increase in the total phenolic content and increments in the antioxidant capacity of the extracts has been observed, the flavonoid profile of the extracts was not altered. As the extraction yields of supercritical fluid extraction can be low, the use of ultrasound is an efficient way to improve mass transfer mechanisms and the kinetics [41]. Ultrasound has therefore been proposed as a tool of increasing the yield or to decrease the extraction time by compression/decompression, acoustic streaming and cavitation phenomenon.

### 2.5. Microwave Extraction

The effects of microwave power and extraction time on the yield and composition of essential oil from oregano by solvent-free microwave extraction was studied by several researchers.

Solvent-free microwave extraction of essential oil from *Origanum vulgare* L. was performed by Bayramoglu and co-workers [42]. The effects of microwave power and extraction time on the yield and composition of the product were investigated. Solvent-free microwave extraction of *Origanum vulgare* L. was compared with conventional hydrodistillation in terms of process time, yield, composition and physical properties of the essential oil obtained. Significantly higher essential oil yields were attained by solvent-free microwave extraction at higher power levels compared to conventional hydrodistillation. Required extraction time decreased significantly, whilst the compositions of the essential oil obtained by both methods were similar. Thymol (650–750 mg/mL) was identified as the major aroma compound.

### 2.6. Clevenger Distillation

Some studies report on steam distillation to obtain essential oil from different species. As the steam rises through the container holding the plants, it causes the oil membranes to open, releasing the essential oils. This process prevents the membrane that surrounds the oil from bursting, which ruins the oil. The oil evaporates and is taken with the steam up through the tube attached at the top of the container. To our knowledge, there are no studies connected to oregano oleoresin extraction and fractionation to separate antioxidant compounds. The chemical composition of the essential oils was analyzed via GC-MS technique and carvacrol and *p*-cymene were identified as the major compounds and additionally, according to the MIC test results, the oregano and savory essential oil showed a perfect antimicrobial activity against 19 test microorganisms with a MIC value of <0.195 µg/mL [43]. 

### 2.7. Identification and Quantitative Determination of Components

High resolution and the ability to provide precise and accurate qualitative and quantitative data established gas-chromatography (GC) coupled with mass spectrometry (MS), i.e., GC-MS analyses as a valuable means for taxonomic research of plants. Several phenolic compounds have been found in plant materials using different detectors, such as the coulometric, electrochemical and photodiode arrays, as well as ultraviolet-visible spectrophotometry and low-resolution mass spectrometry [44]. 

However, techniques employing high-resolution mass spectrometry are still under intense examination, thus the data on identification of the phenolic profile by high-resolution mass spectrometry are still relatively limited. These methods, comprising linear ion trap (LIT) mass spectrometry—which provides single stage mass analysis that supplies molecular mass information, tandem mass analysis (MS/MS) and multi-staged tandem mass spectrometry (MS^n^) that provide structural information—have been demonstrated as a reliable tool for the structural elucidation of unknown compounds in complex mixtures such as the total plant extracts. Flavonoids in the oregano extracts were analyzed using a high-pressure liquid chromatography-diode array detection-electrospray ionization/mass spectrometry (HPLC-DAD-ESI/MS) screening method [45]. 

## 3. Results

Luteolin-7-*O*-glucoside, apigenin 7-*O*-glucoside, phloridzin, taxifolin, eriodictyol, scutellarein, luteolin, quercetin, naringenin, pinocembrin and galangin were identified as the major compounds [46]. The report of Ze-Lin et al. [46] presents the identification and quantification of 23 flavonoids in Mexican oregano. A recent investigation reported on phenolic metabolites that have beneficial effects on human health, including inhibition of mutagenesis and carcinogenesis. To identify the phenolic profiles in different oregano extracts, HPLC analysis was carried out. In total, five major phenolic metabolites were identified; rosmarinic acid, caffeic acid, coumaric acid, protocatechuic acid and quercetin [47].

The volatile aroma components from extracts were analyzed by GC–MS. The GC-FID and GC-MS analysis of essential oil from oregano leaves (*Origanum compactum*) resulted in the identification of 46 compounds, representing more than 98% of the total composition. Carvacrol was the predominant compound (36.46%), followed by thymol (29.74%) and p-cymene (24.31%). Serial extractions with petroleum ether, ethyl acetate, ethanol and water performed on aerial parts of *Origanum compactum* produced extracts with various chemical species: polyphenols (gallic acid equivalent 21.2 to 858.3 g/kg), tannins (catechin equivalent 12.4 to 510.3 g/kg), anthocyanins (cyanidin equivalent 0.38 to 5.63 mg/kg) and flavonoids (quercetin equivalent 14.5 to 54.7 g/kg) [48].

In the work of Stanojević and co-workers [49], qualitative and quantitative composition of the oil was determined by GC-MS and GC-FID spectrometry. The major compound identified was α-thujene, followed by myrcene, α-terpinene, o-cymene, γ-terpinene, thymol and carvacrol.

**Figure 2:**
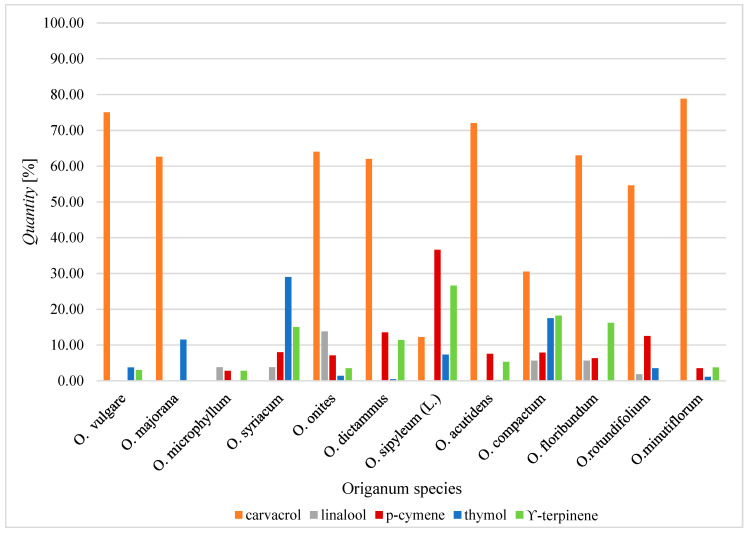
The proportion of the major bioactive compounds of the essential oils in different species of oregano [9].

Figure 2 presents the proportion of the major bioactive compounds of the essential oils in different species of oregano. It can be seen that carvacrol is the most common compound identified in the *Origanum* species. 

Little is known about the antioxidant activity of particular oregano compounds. Flavonoids and phenolic acids from oregano such as carnosic acid, rosmanol and rosmarinic acid, luteolin, hispidulin, apigenin, acacetin, diosmetin, herbacetin, quercetin and naringin have been reported as the compounds with major antioxidant properties. High proportions of these compounds were found in aqueous, methanolic and ethanolic extracts obtained under selected conditions from oregano leaves [50]. Table 1 offers an overview of the quantity of different active compounds in several *Origanum* species in addition to the suitable isolation method. Typical biological activities of certain species are presented in the Table 2. 

Indeed, antiradical activity has been found in aqueous and methanolic extracts of oregano leaves. Ethanolic extracts under selected conditions showed antioxidant activity as well. Even though a variety of flavonoids are known, there is no correlation between compositional data and antioxidant activity. The total phenolic content does not directly forecast the potent stabilization of the phenoxyl radical. The structure of different flavonoids extracted in different conditions will finally define the antioxidant activity of the extract. The number and position of hydroxyl and methoxyl groups in the benzene ring and the possibility of electron delocalization in the double bonds are the most important aspects that influence the final activity of the extracts [73].

In addition, essential oils have also been recognized as substances responsible for antioxidant activity and may represent beneficial alternatives to synthetic antioxidants in food products. The essential oil from different species of oregano can be used alone or in combination with other essential oils, food additives or preservation techniques, to improve the shelf life of food products [42]. Although carvacrol and thymol are primarily known for their antimicrobial activity, some authors have reported on their antioxidant activity [50]. The radical-scavenging activity of carvacrol and thymol, both phenolic compounds, is optimal for blocking alimentary-free radicals, their chain-breaking activity protects against peroxidized foods, and their iron-chelating activity defends the body from the deleterious action of free iron. The dietary effects of oregano extracts on the lipid oxidative stability of meat have also been reported [74].

A variety of methods, classified in two major groups, is used to determine the antioxidant ability. Antioxidant activity/capacity can be monitored by assays with different mechanisms, including hydrogen atom transfer (HAT) and electron transfer (ET). There are also mixed-mode (ET/HAT) assays, generally without distinct boundaries between them. The appropriate selection of technique is based on understanding the principal mechanisms and advantages and drawbacks of the assay. These considerations are important for the valid evaluation of antioxidant properties in desired applications. Among HAT assays, the oxygen radical absorbance assay (ORAC) is the most frequently used. The most recently used ET methods are: the inhibition of the 1,10-diphenyl-2-picrylhydrazyl radical (DPPH), the Trolox equivalent antioxidant capacity (TEAC) method/ABTS radical cation decolorization assay (also known as ABTS assay), the ferric-reducing antioxidant power assay (FRAP), the cupric ion-reducing antioxidant capacity method (CUPRAC) and total phenolic content assay [75].

It has been reported that the antioxidant capacity of extracts from different oregano species is somewhat dependent on the solvents used during their extraction. The antioxidant ability of extracts has been correlated with the flavonoid and phenolic acids yield during the extraction process. Greek oregano (*Origanum vulgare*) is the most recognized species of oregano. Methanolic extracts of *Origanum vulgare* have shown high antioxidant capacity against DPPH, ABTS, FRAP and ORAC [19], which is attributed to the high phenolic content such as rosmarinic acid, eriodictyol, naringenin and epicatechin [76]. Water, methanol, ethyl acetate and hexane extracts contain a high content of rosmarinic, caffeic, chicoric and *p*-coumaric acids. TPC, DPPH, TAC, and RP assays have been applied to confirm their antioxidant ability [31].

Recent literature reports the strong antioxidant ability of methanolic extract of the stem against the DPPH radical (96% at 200 ppm), superoxide anion radical scavenging (61% at 250 ppm) and TAC (634 µM AAE/g of extract). Subsequent HPLC analysis confirmed high amounts of rosmarinic acid, caffeic acid, rutin, gallic acid, quercetin and *p*-coumaric acid in the examined samples. Nevertheless, as reported by Yan et al. [77], the high content of rosmarinic acid analyzed in the hydro-extract of the leaves from *Origanum vulgare* did probably not contribute to the high antioxidant activity determined by the TPC and ORAC assays. Phenolic content, provided as gallic acid equivalents per amount of fresh weight, varied between 79 mg and 147 mg GAE/g DW. Trolox equivalents (TE)/mg of dry weight varied between 1.59 mmol and 3.39 mmol TE/g DW. Thus, no correlation was found between the rosmarinic acid content and the antioxidant capacity measured by ORAC, which may indicate that other compounds are acting as antioxidant agents. In contrast, the study of Gonçalves et al. [24] reports that the high quantity of rosmarinic acid (23.53 mg/g of dry extract) and the presence of other active compounds, including epicatechin, in the same type of extracts contributed to the high antioxidant capacity (as determined by DPPH, ABTS and FRAP). Some authors relate the antioxidant activity of *Origanum vulgare* with the presence of rosmarinic, chicoric and caffeic acid [31]. The total phenolic content and corresponding antioxidant activity of extracts from different oregano species is given in Table 2.

Furthermore, eriodictyol and naringenin were also found in the methanolic extract of *Origanum vulgare* leaves, which exhibited high TPC (430 µg of GAE/mg of DWE) and a positive relationship with the ORAC value (~11 mmol TE/mg DWE) [19].

A large content of flavonoids luteolin-7-*O*-glucoside and apigenin-7-*O*-glucoside attained by accelerated solvent extraction with methanol is assumed to contribute to the high TPC and the FRAP value [18]. A study of Balkan et al. [78] reports a connection between the TPC and DPPH scavenging activity with the presence of eriodictyol, apigenin and caffeic acid in the aqueous extract of *Origanum vulgare* [76]. As a significant decrease of the oxidative processes in a fat substance can be attained by adding a low concentration of oregano extracts, the single phenolic compounds cannot always be quantitatively determined by means of traditional chromatographic techniques, such as GC and HPLC. 

In the work of Vichi et al, a sensitive analytical method is proposed to determine the presence of phenolic compounds in animal fat samples [79]. Determination was achieved by the assessment of the antioxidant activity of the polar fraction, obtained by solid phase extraction, in order to separate and concentrate the phenolics from lipidic substances.

Essential oils, in general, have shown lower antioxidant activity than artificial antioxidants even when using the essential oils in higher concentrations. Quiroga and co-workers reported that [80] diverse chemical compositions of oregano essential oils reflected different antioxidant activities. In that research, oregano essential oil showed a higher thymol content and higher radical scavenging and antioxidant activity when added to canola oil. Namely, increasing the concentration of a particular molecule with high antioxidant potential in a fraction obtained from the essential oil should increase the antioxidant power in that fraction compared with the antioxidant activity of the essential oil. This may be contributed to the antioxidant synergy among the compounds present in both extracts. When the antioxidants are combined, antioxidant synergy occurs. Networking antioxidants can recycle one another from their radicalized forms back into their active antioxidant forms. By this process of mutual regeneration, networking antioxidants enhance and extend one another’s capacities, working within and around the mitochondria where most of the oxidation occurs. Flavonoids and phenolic acids found in different oregano species are listed in Table 3.

Antimicrobial activities of the essential oils derived from plants have gained the attention of researchers as they might represent an alternative to the increasing resistance of conventional antibiotics against pathogen infections. Many researchers have been oriented towards the antimicrobial activity of essential oils against a wide range of microorganisms, and particularly against common food pathogens [91] as presented in Table 4. Oregano essential oil was one of the first natural antiseptics used, proving a good bactericide effect against gram-positive and gram-negative bacteria as well as showing both antifungal and antioxidant activity. The main advantage of these natural products is that they do not enhance antibiotic resistance, as seen with the long-term use of synthetic antibiotics. On the other hand, the ability of plant essential oils to protect foods against pathogenic and spoilage microorganisms with the benefit of being natural products and thus more acceptable by the consumers, has been reported by several researchers [92].

The essential oil of *Origanum vulgare* had substantial antimicrobial activity against 10 bacteria, 15 fungi and a yeast species [105]. Carvacrol, *p*-cymene, α-terpinene and thymol have been identified as the major compounds of oregano essential oil which is recognized as a natural antimicrobial agent with a strong potential for food preservation. Their antimicrobial effect against food-borne pathogens including gram-positive bacteria such as *Listeria monocytogenes* and gram-negative *Escherichia coli* is initialized by making the cell membrane permeable due to its impregnation in the hydrophobic domains. This effect is greater against gram-positive bacteria such as *Listeria monocytogenes*. Gram-negative *Escherichia coli* is a large group of bacteria found in the environment, foods and intestines of people and animals, and for that reason, is a subject of several studies. Oregano essential oil has antioxidant properties effective in retarding the process of lipid peroxidation in fatty foods, and scavenging free radicals. In this perspective, the present subchapter analyzes and discusses the state of the actual and potential uses of oregano essential oil as antimicrobial and antioxidant food additives. The intense aroma of oregano essential oil influences organoleptic characteristics of food products. A lot of research has been dedicated to settle the compromise between the beneficial antimicrobial and antioxidant properties and minimizing the undesired sensory effects. 

The most relevant constituents of the essential oils are volatile. That fact needs to be considered in the research of functional packaging materials to obtain an efficient method of incorporation. Recent research is oriented towards the development of active packaging that simultaneously provides protection against oxidation and microbial deterioration of food products [106]. Antioxidant and antimicrobial properties of ethylene vinyl alcohol copolymer films were investigated based on the release of oregano essential oil and green tea extract components by Muriel-Galet et al. [107]. Polymer films are manufactured by a casting procedure which can be considered as a simulation of industrial film coating by printing procedures. Release kinetics depend on the affinity between active agents and food simulants.

Antimicrobial washing treatments are used to eliminate pathogenic bacteria that inhabit fresh produce surfaces and to prevent spread in the fresh produce processing environment. Plant-derived compounds gain interest due to their antimicrobial activity and their consumer-friendly nature. The effect of single or combined chemical and natural antimicrobial interventions on *Escherichia coli* O157:H7 has been studied, where the total microbiota and color was tested after treatment and storage of fresh lettuce and spinach. Washing lettuce samples with oregano for 2 min resulted in 2.1 log CFU/g reduction of *E. coli* O157:H7. The results indicated that vinegar, lactic acid or oregano aqueous extract alone or in combination, as alternative washing solutions to chlorine, may be effectively used to control *E. coli* O157:H7 and sustain the acceptable appearance of fresh cut spinach and lettuce [108].

A perspective application of the plant extracts may be in the process of food preparation. Marination before cooking is a common culinary technique used to create tenderness and enhance flavor in foods, particularly in meats. Marinades as wine formulations containing essential oils from oregano were evaluated for inhibitory activity against the foodborne pathogens *Escherichia coli* O157:H7 and *Salmonella enterica*. The high activity of oregano essential oils in red wine was reduced in *E. coli*, but not in *Salmonella*. Apparently, the essential oils in red wine can be an effective antimicrobial in food, but the addition of phytochemicals to the treatment solution or their native presence in the food itself, could harmfully influence the antimicrobial activity and should be subjected to the additional examination [109].

The effects of oregano extracts on the oxidative, microbiological and sensory stability of sheep burgers packed in a modified atmosphere were tested with the aim of replacing the synthetic antioxidant BHT with oregano natural extract [55]. The presence of the natural extract prevented the loss of sensory qualities by reducing lipid and protein oxidation. The changes in off-odor were consistent with the microbiological stability which has been limited to 10 days.

## 4. Discussion

Essential oils have largely been used for their antibacterial, antifungal and insecticidal activities. The potential use of oregano essential oils as natural antimicrobials and antioxidants has been reported in meat, fish and vegetable products [110]. The literature also provides the possibility of finding a number of potential synergisms to improve the essential oil usefulness. 

Skandamis and Nychas reported that oregano essential oil delayed microbial growth and suppressed the final counts of the spoilage microorganisms. Oregano essential oil delayed glucose and lactate consumption aerobically as well as under atmosphere consisted from: 40% CO_2_/ 30% N_2_/ 30% O_2_; or CO_2_ [111]. Mejlhom and Dalgaard reported the antimicrobial effect of essential oils on the seafood spoilage microorganism *Photobacterium phosphoreum* in liquid media and fish products [112].

Skandamis et al. [111] researched the effect of oregano essential oil on *Escherichia coli* O157:H7. An eggplant salad, a traditional Greek appetizer, was inoculated with *Escherichia coli* O157:H7 and supplemented with different concentrations of oregano essential oil (0.0, 0.7, 1.4, and 2.1% vol/wt). Afterward, it was stored at different temperatures. It was found out that oregano essential oil delayed microbial growth and suppressed the final counts of the spoilage microorganisms [111].

Gutierrez et al. suggest that oregano might be a suitable decontamination alternative to chlorine for ready-to-eat carrots [76]. 

Fasseas et al. [113] investigated the antioxidant activity in meat treated with oregano. The results indicated that the essential oil treatments decreased the oxidation, while the heat treatment and storage time significantly affected the antioxidant activity of the meat [113]. The effect of light salting, modified atmosphere packaging and addition of oregano essential oil showed a positive effect on the shelf-life of sea bream (*Sparus aurata*) [114]. The essential oil of *Origanum vulgare* has been evaluated in patients infected with enteric parasites such as *Blastocystis hominis*, *Entamoeba hartmanni*, and *Endolimax nana*. In 7 out of 11 patients that were positive for *Blastocystis hominis*, improvement was observed [115]. Initial studies with the essential oil from *Origanum vulgare* and *Origanum compactum* have shown a mild effect in vitro on *Plasmodium falciparum* [116]. 

The activity of *Lippia berlandieri* has been assessed on protozoans. In vitro treatment of *Giardia intestinalis* trophozoites with oregano extracts in ethanol [117] or in water [118] causes irreversible structural damage to the nucleoskeleton. This antiparasitic activity was more potent than tinidazole, a traditional giardiasis drug. The essential oils of *Lippia* spp. have been evaluated in a wide range of biological contexts in traditional medicine [119]. 

## 5. Conclusion and Future Perspectives

The extracts of *Origanum vulgare* possess compounds with antimicrobial and antifungal properties, as well as antioxidant activity, and therefore can be used as a natural preservative ingredient in food and/or pharmaceutical industry. It is known that most of their properties are due to the essential oils and other secondary plant metabolites. Essential oils enhance the production of digestive secretions, stimulate blood circulation, exert antioxidant properties, reduce levels of pathogenic bacteria and may enhance immune status. They have been used for several applications in pharmaceutical, cosmetic, agricultural and food industries. Extraction could be carried out by various techniques. Innovative methods avoid shortcomings of conventional techniques like chemical alteration risk, long extraction time and high-energy input. A complex mixture of flavonoids and phenolic acids is responsible for the potential bioactive properties and promising role as an alternative treatment in several illnesses. Interestingly, most of the studies are based on in vitro approaches, limiting its extrapolation to human health. A better knowledge of the mechanism of action and effects of individual compounds would be useful to formulate mixtures of compounds to enhance efficiency. 

## Figures and Tables

**Table 1:** Composition of essential oils from different oregano species.

Species	Active Compound	Quantity %	Type of Isolation	References
*O. majorana*	carvacrol	62.6	conventional steam distillation	[51]
	thymol	11.5		
	phenol	8.2		
*O. vulgare*	camphene	0–1	conventional steam distillation subcritical water extraction	[52,53]
	thymol	3.7		
	carvacrol	75		
	γ-terpinene	2–3		
	linalyl-acetate	0–0.5		
	myrcene	0–0.05		
	terpinen-4-ol	0–2.2		
	caryophyllene	0.9		
	π-cymene	2.1–3.7		
*O. microphyllum*	cis-sabinene	29.8	conventional steam distillation	[54]
	sabinene	20.6		
	trans-sabinene hydrate	14.9		
	γ-terpinene	2.8		
	*p*-cymene	2.8		
	linalool	3.8		
*O. syriacum*	thymol	24–29	hydrodistillation steam distillation	[55,56]
	cis-sabinene hydrate	18–20		
	γ-terpinene	13–15		
	*p*-cymene	5–8		
	terpinen-4-ol	4–8		
*O. onites*	carvacrol	64	hydrodistillation steam distillation	[55,56]
	α-pinene	1.0		
	thymol	1.4		
	myrcene	1.6		
	linalool	13.8		
	*p*-cymene	7.1		
	γ-terpinene	3.5		
	α-terpinene	1.4		
*O. dictamnus*	carvacrol	62	steam distillation	[57]
	thymol	0.44		
	p-cymene	13.49		
	γ-terpinene	11.41		
	r-terpinene	1.88		
*O. sipyleum* (L.)	γ-terpinene	10.8–26.6	steam distillation	[58]
	thymol	0.23–7.3		
	carvacrol	0.82–12.2		
	*p*-cymene	3.7–36.6		
*O. acutidens*	carvacrol	72	hydrodistillation	[59]
	*p*-cymene,	7.5		
	γ-terpinene	5.3		
	borneol	0.9		
	thymol	0.2		
*O. compactum*	carvacrol	30.53	hydrodistillation	[59]
	p-cymene	7.89		
	α-terpinene	2.59		
	thymol	17.5		
	γ-terpinene	18.2		
*O. floribundum*	carvacrol	63	hydrodistillation	[59]
	*p*-cymene	6.3		
	linalool	5.6		
	γ-terpinene	16.2		
*O. rotundifolium*	carvacrol	54.6	hydrodistillation	[60]
	*p*-cymene	12.5		
	borneol	5.9		
	thymol	3.5		
	linalool	1.8		
	terpinene-4-ol	1.5		
	thymohydroquinone	1.14		
	β-Caryophyllene	1.09		
	germacrene D	1.08		
	linalyl acetate	1.07		
*O. minutiflorum*	carvacrol	78.8	hydrodistillation	[61]
	γ-terpinene	3.7		
	*p*-cymene	3.5		
	thymol	1.1		
	α-pinene	1.3		

**Table 2:** Typical biological activities of certain oregano species.

Species	Biological Activity
*O. majorana*	antioxidant [51] antiproliferative [51] antibacterial [51] cytotoxic [62,63]
*O. vulgare*	antiproliferative activity on tumor cells of Hela [10,17] antiradical [51] antifungal [52,53] antihyperglycaemic [63] antithrombin [64] antioxidant [65,66] antimicrobal [67,68]
*O. microphyllum*	antioxidant [54]
*O. syriacum*	antibacterial [55,56] antimycotic [55,56] antioxidative [55,56]
*O. onites*	antibacterial [55,56] antimycotic [55,56] antioxidative [55,56] antispasmodic [69] antibacterial [70,71] antifungal [72]
*O. dictammus*	antibacterial [57] antifungal [57]
*O. sipyleum* (L.)	effective on respiratory and gastrointestinal disorders [58]
*O. acutidens*	antibacterial [59] antifungal [59] antioxidant [59]
*O. compactum*	antioxidant [59] antifungal [59] cytotoxic [59]
*O. floribundum*	against diarrhoea and other digestive disorders [59]
*O. rotundifolium*	antibacterial [60]
*O. minutiflorum*	antibacterial [61]

**Table 3:** Flavonoids and phenolic acids found in different oregano species [76].

Oregano Species	Components	Extraction Solvent Used	Ref.
*Coleus aromaticus*	acids: − caffeic, − gallic acids; − *p*-coumaric − rosmarinic, quercetin rutin	Methanol	[81]
*Eryngium foetidum*	acids: − ferulic − gallic − *p*-coumaric − protocatechui − sinapic − syringic	Aqueous, methanol	[82]
*Lippia alba*	apigenin-7-*O*-diglucuronide chrysoeriol-7-*O*-diglucuronide tricin-7-*O*-diglucuronide luteolin-7-*O*-glucuronide	Aqueous	[83]
*Lippia graveolens*	naringenin rosmarinic acid	Methanol	[84]
	cirsimaritin eriodictyol hispidulin naringenin	Methanol	[19]
*Origanum dictamnus*	Aqueous: caffeic, protocatechuic and rosmarinic acids Methanolic: gallic, caffeic, ferulic and rosmarinic acids.	Sequentially with hexane, acetone and methanol; Aqueous	[85]
	Acids: − caffeic − rosmarinic − vanillic catechin epicatechin genistein	Aqueous	[25]
*Origanum glandulosum*	caffeic acid luteolin glucoside	Methanol, previously defatted with *n*-hexane	[85]
*Origanum majorana*	Acids: − caffeic − rosmarinic apigenin rutin	Methanol microwave-assisted	[86]
	acids: − rosmarinic − caffeic	Aqueous, methanol	[87]
	cirsimaritin eriodictyol hispidulin naringenin rosmarinic acid	Methanol	[19]
	apigenin epigallocatechin quercetin rosmarinic acid	Methanol	[21]
	acids: − chlorogenic − ferulic − *p*-coumaric − *p*-hydroxybenzoic − protocatechuic − rosmarinic − syringic quercetin	Ethanol	[88]
*Origanum microphyllum*	acids: − caffeic − *p*-Hydroxybenzoic − protocatechuic − syringic naringenin	Aqueous	[89]
*Origanum vulgare*	rosmarinic acid	Methanol	[77]
	(−)-epicatechin rosmarinic acid	Methanol	[31]
	acids: − caffeic − chicoric − *p*-coumaric − rosmarinic	Water, methanol, ethyl acetate, hexane	[31]
	eriodictyol naringenin rosmarinic acid	Methanol	[19]
*Origanum vulgare*	acids: − caffeic − rosmarinic apigenin-7-*O*-glucoside luteolin-7-*O*-glucoside	Methanol	[90]
	apigenin caffeic acid eriodictyol kaempferol	Aqueous	[76]
*Thymbra capitata*	Ethyl acetate: − taxifolin di-*O*-glucoside − thymusin. Ethanol: − taxifolin di-*O*-glucoside − rosmarinic acid	Ethyl acetate, ethanol	[17]

**Table 4:** Antimicrobial activities of different oregano species.

Species	Bacteria Inhibition	Ref.
*O. minutiflorum*	*Campylobacter spp.* *Total aerobic mezophyl bacteria* *Rhipicephalus turanicus* *Labiatae family* *E. Coli* *S. Aureus* *L. Monocytogenes* *S. Sonnei* *P. Aeruginosa* *Helicobacter pylori*	[93,94]
*O. vulgare*	*Gram-positive* *Gram-negative bacteria, listeria monocytogenes* *Staphylococcus aureus* *Escherichia coli* *Bacillus cereus* *Stafilococcus aureus* *Lysteria monocytogenes* *Salmonella* *Typhimurium* *Trypanosoma cruzi*	[66,95,96,97,98,99,100,101,102]
*O. majorana*	*Bacillus subtilis* *Clostridium botulinum* *Escherichia coli* *Listeria monocytogenes* *Salmonella typhimurium* *Staphylococcus* spp. *Spodoptera littoralis*	[95,103,104]
*O. compactum*	*Mayetiola destructor*	[13,18]
*O. syriacum*	*Phytophthora infestans*	[101]
*O. onites*	*Plant pathogenic bacteria*	[71]
